# Grape/Blueberry Anthocyanins and Their Gut-Derived Metabolites Attenuate LPS/Nigericin-Induced Inflammasome Activation by Inhibiting ASC Speck Formation in THP-1 Monocytes

**DOI:** 10.3390/metabo14040203

**Published:** 2024-04-03

**Authors:** Inken Behrendt, Isabella Röder, Frank Will, Gabriela Michel, Elvira Friedrich, Daniela Grote, Zoe Martin, Hanna Pauline Dötzer, Mathias Fasshauer, Martin Speckmann, Sabine Kuntz

**Affiliations:** 1 Institute of Nutritional Science, Justus-Liebig-University Giessen, 35390 Giessen, Germany; elvira.friedrich@ernaehrung.uni-giessen.de (E.F.); grote.d@outlook.de (D.G.); martin.zoe@gmx.de (Z.M.); hanna.doetzer@t-online.de (H.P.D.); mathias.fasshauer@ernaehrung.uni-giessen.de (M.F.); sabine.kuntz@nutr.jlug.de (S.K.); 2 Department of Beverage Research, Hochschule Geisenheim University, 65366 Geisenheim, Germany; isabella.roeder@googlemail.com (I.R.); frank.will@hs-gm.de (F.W.); 3 Institute for Clinical Immunology, Transfusion Medicine and Hemostaseology, Justus-Liebig-University Giessen, 35392 Giessen, Germany; gabriela.michel@immunologie.med.uni-giessen.de (G.M.); martin.speckmann@immunologie.med.uni-giessen.de (M.S.); 4 Flow Cytometry Core Facility, Department of Medicine, Justus-Liebig-University Giessen, 35392 Giessen, Germany

**Keywords:** anthocyanins, gut-derived metabolites, NLRP3 inflammasome, ASC specks, grapes, blueberries

## Abstract

Inflammasomes are multi-protein complexes, which are formed in response to tissue injury, infections, and metabolic stress. However, aberrant inflammasome activation has been linked to several inflammatory diseases. Anthocyanins have been reported to attenuate NLR family pyrin domain-containing 3 (NLRP3) inflammasome activation, but the influence of grape/blueberry anthocyanins and especially their gut-derived metabolites on NLRP3 inflammasome activation in human monocytes remains unclear. Therefore, human leukemic monocytes (THP-1 cells, Tohoku Hospital Pediatrics-1 cells) were preincubated with different concentrations of grape/blueberry anthocyanins, homovanillyl alcohol, or 2,4,6-trihydroxybenzaldehyde (THBA) before the NLRP3 inflammasome was activated by lipopolysaccharide and/or nigericin. Apoptosis-associated speck-like protein containing a CARD (ASC) speck formation, as well as ASC and NLRP3 protein expression, were determined using flow cytometry. Caspase-1 activity was measured in cultured cells, and pro-inflammatory cytokine secretion was determined using enzyme-linked immunosorbent assays. Anthocyanins and their metabolites had no effect on ASC or NLRP3 protein expression. However, THBA significantly inhibited ASC speck formation in primed and unprimed THP-1 monocytes, while caspase-1 activity was significantly declined by grape/blueberry anthocyanins. Furthermore, reduced inflammasome activation resulted in lower pro-inflammatory cytokine secretion. In conclusion, our results show for the first time that grape/blueberry anthocyanins and their gut-derived metabolites exert anti-inflammatory effects by attenuating NLRP3 inflammasome activation in THP-1 monocytes.

## 1. Introduction

Anthocyanins are water-soluble secondary plant metabolites, which represent a subclass of flavonoids [1]. They possess a broad range of biological activities and several epidemiological [2,3,4,5], as well as experimental [6,7], studies indicate their beneficial health effects especially on cardiovascular diseases. In Europe, mean dietary anthocyanin intake is reported to range from 19 to 65 mg/d, where berries, grapes, and wine are the main dietary sources [8]. However, in contrast to other flavonoids, absorption of anthocyanins is low [9]. Therefore, most of the ingested anthocyanins reach the colon, where they are extensively metabolized by the gut microbiota into low-molecular-weight metabolites [7,10,11]. These metabolites may be absorbed in the colon by the monocarboxylate transporter-1 [12], thus the gut microbiota plays a crucial role in anthocyanin bioavailability [7,11]. In addition, anthocyanins are unstable at basic pH values and spontaneous degrade into aldehydes and phenolic acids, such as 2,4,6-trihydroxbenzaldehyde (THBA, phloroglucinaldehyde) and 3,4-dihydroxybenzoic acid (protocatechuic acid) in the gastrointestinal tract [7,13]. Hence, gut-derived metabolites can derive from the human gut microbiota or spontaneous anthocyanin degradation. THBA is a degradation product, which results from the A-ring, thus THBA could be formed by each parent anthocyanin independent of B-ring substitution [14]. Therefore, THBA is found at much higher plasma concentrations compared to its parent anthocyanins [15,16]. In this context, using UHPLC–MS/MS analyses, we have recently shown in a randomized, placebo-controlled, crossover trial that THBA was significantly increased in plasma after long-term consumption of an anthocyanin-rich grape/blueberry juice, while plasma concentrations of parent anthocyanins were unaltered [17]. In addition, homovanillyl alcohol (HVA) was significantly increased in feces samples after consumption of the grape/blueberry juice (unpublished data). Furthermore, parent anthocyanins have a short half-life compared to their low-molecular-weight metabolites [10]. Therefore, it is assumed that observed beneficial health effects of dietary anthocyanins are mainly mediated by persistent high metabolite concentrations [18].

Inflammasomes are multi-protein complexes, which can be formed by myeloid cells in response to tissue injury, infections, and metabolic stress as part of the innate immune response [19,20]. However, aberrant inflammasome activation has been linked to several inflammatory diseases such as atherosclerosis, diabetes mellitus type 2, and neurodegenerative diseases [21]. Canonical NLR family pyrin domain-containing 3 (NLRP3) inflammasome activation leads to the formation of a large multimeric ASC speck, comprising NLRP3, the adaptor molecule apoptosis-associated speck-like protein containing a CARD (ASC), and pro-caspase-1. Upon inflammasome assembly, auto-catalytic activation of caspase-1 takes place, resulting in the maturation and release of the inflammatory cytokines IL-1β and IL-18. Furthermore, activation of caspase-1 causes pyroptosis, an inflammatory form of programmed cell death [22]. Thus, inhibition of the NLRP3 inflammasome may exert beneficial health effects in the prevention and treatment of inflammatory diseases. Therefore, searching for new approaches to prevent NLRP3 inflammasome activation is crucial. Anthocyanins and their gut-derived metabolites are known for their anti-inflammatory and anti-oxidative properties [23,24,25]. In addition, anthocyanins have been reported to attenuate NRLP3 inflammasome activation in several in vitro and in vivo studies. For instance, malvidin supplementation diminished NLRP3 inflammasome activation in mice with LPS-induced acute liver injury [26]. Similarly, malvidin inhibited activation of the NLRP3 inflammasome in mice with acute kidney injury and human renal tubular epithelial cells [27]. However, the influence of grape/blueberry anthocyanins and especially their gut-derived metabolites on NLRP3 inflammasome activation in human monocytes remains unclear. Therefore, the present study aimed to determine the potential of grape/blueberry anthocyanins and their gut-derived metabolites homovanillyl alcohol (HVA) and THBA to attenuate NLRP3 inflammasome activation. In summary, to the best of our knowledge, our results show for the first time that grape/blueberry anthocyanins and their gut-derived metabolites exert anti-inflammatory effects by attenuating NLRP3 inflammasome activation in THP-1 monocytes.

## 2. Materials and Methods

### 2.1. Preparation and Characterization of the Powdered Anthocyanin-Rich Grape/Blueberry Extract

The anthocyanin-rich grape/blueberry extract (GBE) was produced at Geisenheim University (Department of Beverage Research, Geisenheim, Germany) from an anthocyanin-rich grape/blueberry juice. Briefly, the juice was made from 80% red grape juice (grape variety Accent (Hochschule Geisenheim University, Geisenheim, Germany)) blended with 20% bilberry juice (Heidelbeersaft blank BIO; Bayernwald KG, Hengersberg, Germany). The juice was loaded onto a pilot column (Kronalab Chromatographie und Labortechnik GmbH, Sinsheim, Germany) filled with SP70 Sepabeads^®^ absorber resin (Resindion S.r.l., Binasco, Italy), and water-soluble juice constituents like sugars, organic acids, and minerals were washed out with distilled water. Afterwards anthocyanins and colorless polyphenols were eluted with ethanol (Merck GmbH, Darmstadt, Germany). The ethanolic fraction was concentrated using rotary evaporation (Hei-Vap Industrial 0.1.6; Heidolph Instruments GmbH & Co. KG, Schwabach, Germany), and the resulting anthocyanin-rich extract was spray dried (B-290 Mini Spray Dryer; Büchi Labortechnik GmbH, Essen, Germany) with maltodextrin (DE17-19; Applichem GmbH, Darmstadt, Germany) as the carrier agent. The powdered extract was analyzed after membrane filtration (0.45 μm) for total phenolics, and anthocyanins were analyzed using HPLC–PDA/ESI–MS as previously described [28]. A representative LC–MS chromatogram of the powdered anthocyanin-rich grape/blueberry extract is shown in Figure S1. Quantitation was carried out in duplicate using peak areas detected at 520 nm and based on external calibration via the reference substance cyanidin-3-glucoside (0.1–100 mg/L; linearity of calibration, r^2^ = 0.9999). For cyanidin-3-glucoside, the limit of detection was 0.01 mg/L and the limit of quantitation was 0.04 mg/L. Anthocyanins were identified using mass spectra and literature data.

### 2.2. THP-1 Cell Culture

THP-1 cells are derived from a 1-year-old patient with acute monocytic leukemia and are reported to be a reliable model for NLRP3 inflammasome activation [22,29]. The THP-1 cell line was obtained from Leibniz Institute DSMZ-German Collection of Microorganisms and Cell Cultures (Braunschweig, Germany). Monocytic THP-1 cells were cultured in complete media RPMI-1640 GlutaMAX™ (Invitrogen GmbH, Darmstadt, Germany) supplemented with 7.5% fetal bovine serum (Invitrogen GmbH) and 10 mM Hepes (Invitrogen GmbH). Cells were kept at 37 °C in a humidified incubator at 5% CO_2_, and cell density was maintained between 2 × 10^5^ and 1 × 10^6^ cells/mL. THP-1 cells can be cultured up to 3 months without a change in cell behavior [30] and were used between passage 9 and 18.

### 2.3. Cell Viability and Cytotoxicity

THP-1 cells (8 × 10^5^ cells/mL) were seeded in 48-well plates and subjected to various concentrations of grape/blueberry anthocyanins or their gut-derived metabolites [17]. The molecular structures of the gut-derived metabolites HVA and THBA are shown in Figure S2. The GBE was freshly prepared each time at a concentration of 1 mg/mL. For HVA (purity 99%, CAS No.: 2380-78-1; Sigma-Aldrich, Taufkirchen, Germany) and THBA (purity ≥ 97%; CAS No.: 487-70-7; Sigma-Aldrich), stock solutions of 10 mM were prepared. Treatment solutions were sterile-filtered, and THP-1 cells were incubated with the indicated concentrations for 24 h. The cytotoxic effects of each treatment solution were assessed using flow cytometry. Therefore, THP-1 cells were stained with Guava^®^ ViaCount^TM^ Reagent (Merck GmbH) and incubated for 10 min at room temperature in the dark. A total of 1000 events were acquired on a Guava^®^ Muse^®^ Cell Analyzer (Merck GmbH), and cell viability was determined. The representative gating strategy is shown in Figure S3.

### 2.4. NLRP3 Inflammasome Activation

To examine the influence of grape/blueberry anthocyanins and their gut-derived metabolites on the NLRP3 inflammasome, TPH-1 monocytes were preincubated with different concentrations of the GBE, HVA, or THBA for 24 h before the NLRP3 inflammasome was activated. After preincubation with grape/blueberry anthocyanins or their gut-derived metabolites, the medium was replaced and THP-1 monocytes were either left untreated or primed with 10 ng/mL LPS (*Escherichia coli* 0111: B4, Sigma-Aldrich) in serum-free media for 4 h before the inflammasome was activated by adding 10 μM nigericin (Sigma-Aldrich) for further 40 min.

### 2.5. Flow Cytometry

Intracellular ASC speck formation, as well as ASC and NLRP3 protein expression, were determined using flow cytometry. THP-1 monocytes were fixed and permeabilized (Biolegend, Amsterdam, Netherlands) according to the manufacturer’s instructions. Fixed cells were stored in sodium azide-containing buffer at 4 °C until the next day. Then, cells were intracellularly stained with fluorescence-labeled monoclonal antibodies [anti-ASC-PE (Biolegend) and anti-NLRP3-APC (Miltenyi, Bergisch Gladbach, Germany)] or the corresponding isotype controls [mouse IgG1κ (Biolegend) and IgG1-REAfinity^TM^ (Miltenyi)] in the dark at room temperature for 10 or 20 min, respectively. In total, 10,000 cells were acquired on a BD FACSCanto^TM^ II Flow Cytometer (BD Bioscience, Heidelberg, Germany), and flow cytometric data were analyzed with FlowJo software version 10.8.1 (BD Bioscience, Heidelberg, Germany). To exclude debris, cells were first gated using forward light scatter area (FSC-A) versus side scatter area (SSC-A). Next, FSC-A versus FSC-height (FSC-H) was used to perform doublet exclusion. Single cells were then gated for ASC and NLRP3 expression compared to the matching isotype control, and median fluorescence intensity (MFI) was assessed (Figure S4). High ASC-expressing cells were further gated using ASC fluorescence pulse area (ASC-A) and ASC fluorescence pulse width (ASC-W). ASC speck forming cells were selected via the observed reduction in ASC-W due to ASC condensation and the percentage of ASC speck-positive cells was quantified (Figure S5).

### 2.6. Caspase-Glo^®^ 1 Inflammasome Assay

Caspase-1 activity in cultured cells was measured using the Caspase-Glo^®^ 1 Inflammasome Assay (Promega, Walldorf, Germany). Briefly, THP-1 monocytes (5 × 10^4^ cells) were seeded in a white opaque 96-well plate. Cells were either left untreated or the NLRP3 inflammasome was activated as indicated. For the measurement of caspase-1 activity, 100 μL of Caspase-Glo^®^ Reagent was added to each well and luminescence was measured after 60 min on a BioTek Synergy H1 microplate reader (Biotek GmbH, Karlsruhe, Germany).

### 2.7. Enzyme-Linked Immunosorbent Assays (ELISA)

Secretion of the pro-inflammatory cytokines IL-1β and IL-18 into the cell culture supernatant was determined using commercial ELISA kits (Invitrogen) according to the manufacturer’s instructions. Absorbance was measured at 450 nm using a BioTek Synergy H1 microplate reader (Biotek GmbH).

### 2.8. Statistical Analyses

GraphPad Prism version 10.0.3 (GraphPad Software, San Diego, CA, USA) was used for statistical analyses. All experiments were run in duplicate, and results are expressed as means ± SD (standard deviation). Significant differences between treatments were calculated with one-way analysis of variance (ANOVA) followed by Dunnett’s multiple comparisons test. Adjusted *p*-values of <0.05 were considered statistically significant, and adjusted *p*-values are given in all figures.

## 3. Results

### 3.1. Composition of the Powdered Anthocyanin-Rich Grape/Blueberry Extract

In total, the anthocyanin content of the powdered grape/blueberry extract was 61.4 mg/g. Due to the high grape content of the juice that the extract originated from, almost half of the identified anthocyanins were peonidin derivates (about 47%). Peonidin-3,5-diglucoside was the most abundant anthocyanin, followed by malvidin-3,5-diglucoside and delphinidin-3-arabinoside (Table 1). The total phenol content of the powdered grape/blueberry extract was 185 mg/g, with 18 mg/g colorless phenolics.

### 3.2. Effect of Grape/Blueberry Anthocyanins and Their Gut-Derived Metabolites on THP-1 Cell Viability

**Figure 1:**
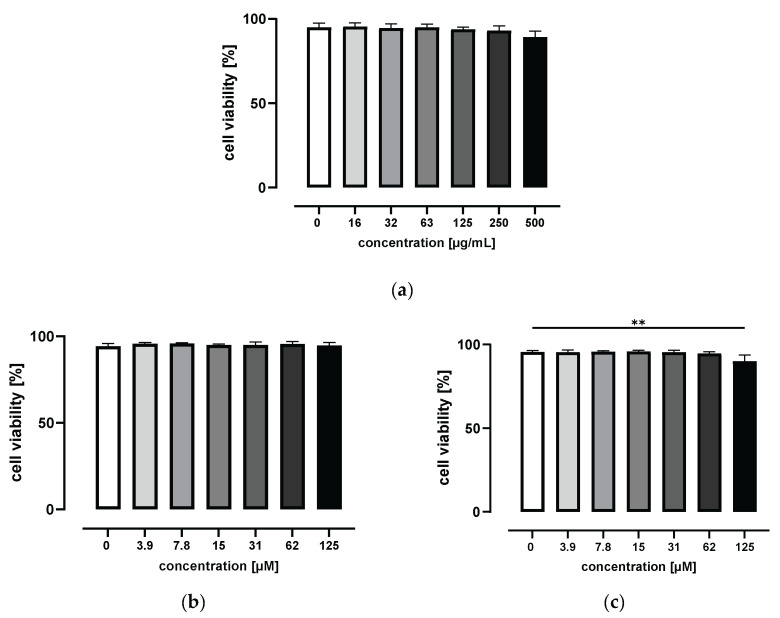
Effect of grape/blueberry anthocyanins and their gut-derived metabolites on THP-1 cell viability. THP-1 monocytes were incubated with the indicated concentrations of (**a**) the GBE, (**b**) HVA, or (**c**) THBA for 24 h before cell viability was measured by flow cytometry. Data are presented as mean ± SD (*n* = 3). Significant differences compared to the untreated control were calculated using one-way ANOVA followed by Dunnett’s multiple comparisons test. ** *p* < 0.01.

To determine the potential cytotoxic effects of grape/blueberry anthocyanins and their gut-derived metabolites on THP-1 cells, THP-1 monocytes were treated with increasing concentrations of the GBE, HVA, or THBA for 24 h before cell viability was measured using flow cytometry. As shown in Figure 1a, incubation with 0 to 500 μg/mL of the GBE for 24 h had no effect on cell viability. In addition, HVA concentrations up to 125 μM were nontoxic to THP-1 monocytes (Figure 1b). In contrast, cell viability was slightly affected by the highest THBA concentration and decreased from 95.5 ± 1.0% for control cells to 90.0 ± 3.8% for THBA-treated cells (*p* < 0.01; Figure 1c). Based on these results, non-cytotoxic GBE concentrations of 15 and 50 μg/mL were used for all subsequent experiments. For gut-derived anthocyanin metabolites, non-cytotoxic concentrations of 1 and 50 μM were selected to represent physiological, as well as pharmacological, doses.

### 3.3. Grape/Blueberry Anthocyanins and Their Gut-Derived Metabolites Have No Effect on ASC and NLRP3 Protein Expression in THP-1 Monocytes

LPS is a toll like-receptor 4 agonist, and priming THP-1 cells with LPS for several hours is reported to result in transcriptional upregulation of several inflammasome components [22]. To investigate the influence of grape/blueberry anthocyanins and their gut-derived metabolites on ASC and NLRP3 protein expression, THP-1 monocytes were preincubated with different concentrations of the GBE, HVA, or THBA before the NLRP3 inflammasome was primed with LPS. ASC and NLRP3 protein expression were determined using flow cytometry, whereas high basal protein expression rates were observed in THP-1 monocytes. However, neither ASC nor NLRP3 protein expression was induced through LPS priming in the present study. In addition, preincubation with grape/blueberry anthocyanins and their gut-derived metabolites had no effect on ASC or NLRP3 protein expression in THP-1 monocytes (Figure S6).

### 3.4. Grape/Blueberry Anthocyanins and Their Gut-Derived Metabolites Attenuate ASC Speck Formation in THP-1 Monocytes

**Figure 2:**
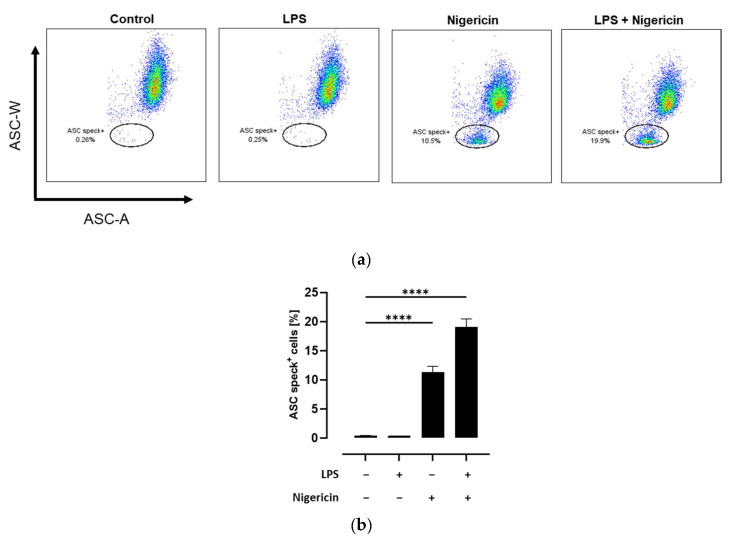
Detection of ASC speck formation in THP-1 cells using flow cytometry. THP-1 monocytes were either left untreated or primed with LPS followed by activation of the NLRP3 inflammasome with nigericin as mentioned in the methods section. Flow analysis was performed and the percentage of ASC speck-positive cells was quantified. Data are presented as (**a**) representative dot plots and (**b**) column bars with mean ± SD (*n* = 3). Significant differences compared to the untreated control were calculated using one-way ANOVA followed by Dunnett’s multiple comparisons test. **** *p* < 0.0001.

ASC speck formation is a hallmark of NLRP3 inflammasome activation. While under resting conditions ASC molecules are diffuse distributed in the cytoplasm, NLRP3 inflammasome activation leads to the formation of a single supramolecular ASC speck. This condensation of ASC molecules could be observed using flow cytometry as a decline in ASC pulse width (ASC-W) [31]. Therefore, the quantification of ASC speck-positive cells using flow cytometry is a sensitive method to determine NLRP3 inflammasome activation on a cellular level [22]. To validate this method, THP-1 monocytes were either left untreated or primed with LPS followed by activation of the inflammasome with the pore-forming toxin nigericin [32]. As expected, no formation of ASC specks could be observed in untreated and LPS-treated THP-1 monocytes. In contrast, nigericin treatment with and without prior LPS priming resulted in a significant increase of ASC speck-positive cells compared to the untreated control (*p* < 0.0001; Figure 2).

**Figure 3:**
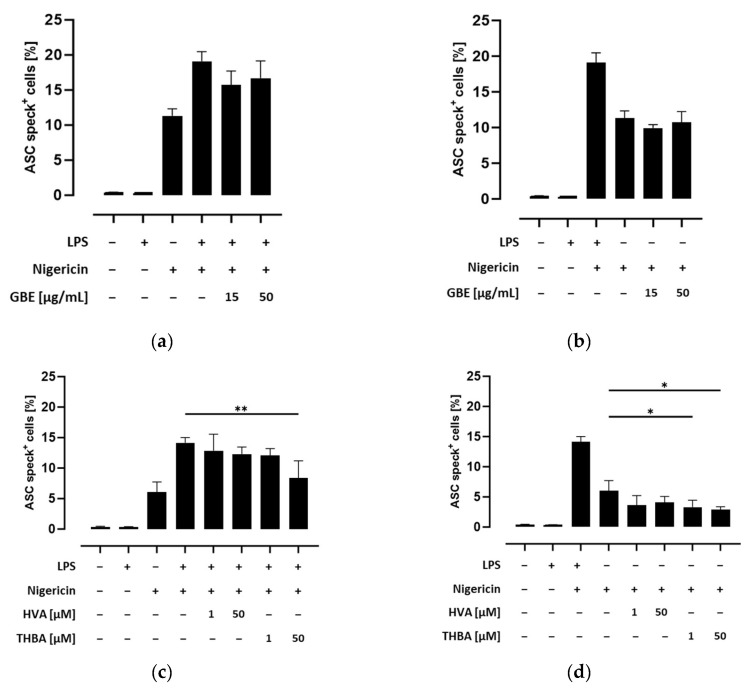
Effect of grape/blueberry anthocyanins and their gut-derived metabolites on ASC speck formation in THP-1 cells. THP-1 monocytes were preincubated with the indicated concentrations of grape/blueberry anthocyanins and their gut-derived metabolites before the NLRP3 inflammasome was activated as mentioned in the methods section. Cells were flow cytometrically analyzed and the percentage of ASC speck-positive cells was quantified. Data are presented as mean ± SD (*n* = 3). Significant differences compared to (**a**,**c**) LPS- and nigericin-stimulated cells or (**b**,**d**) cells treated only with nigericin were calculated using one-way ANOVA followed by Dunnett’s multiple comparisons test. * *p* < 0.05 and ** *p* < 0.01.

After validating this method, we next investigated potential inhibitory effects of grape/blueberry anthocyanins and their gut-derived metabolites towards NLRP3 inflammasome activation. Therefore, THP-1 monocytes were preincubated with different concentrations of the GBE, HVA, or THBA before the NLRP3 inflammasome was activated with LPS and/or nigericin. There was a decline in the percentage of ASC speck-positive cells after preincubation with the GBE (15 μg/mL) in LPS- and nigericin-treated cells. However, after correction for multiple comparisons this effect was no longer statistically significant (Figure 3a). In contrast, no impact of GBE treatment on ASC speck formation in unprimed nigericin-treated cells was observed (Figure 3b). Interestingly, THBA (50 μM) significantly inhibited ASC speck formation in LPS- and nigericin-treated THP-1 monocytes (*p* < 0.01), while HVA had no effect (Figure 3c). However, the percentage of ASC speck-positive cells was decreased by low and high HVA, as well as THBA, doses in unprimed, nigericin-treated THP-1 monocytes, whereas the effects of THBA were still significant after correction for multiple testing (*p* < 0.05; Figure 3d). Taken together, these results indicate that grape/blueberry anthocyanins and their gut-derived metabolites attenuate ASC speck formation in primed and unprimed THP-1 monocytes.

### 3.5. Effect of Grape/Blueberry Anthocyanins and Their Gut-Derived Metabolites on Caspase-1 Activity in THP-1 Monocytes

Upon NLRP3 inflammasome activation, ASC and pro-caspase-1 molecules interact via their respective caspase recruitment domains, leading to the auto-proteolytic activation of caspase-1 [33]. This activation of caspase-1 is usually monitored through western blot analysis of cleaved caspase-1. However, this method does not directly reflect caspase-1 activity. For this reason, in the present study, a bioluminescent, coupled-enzyme assay utilizing a Z-WEHD-aminoluciferin substrate for caspase-1 was used to determine caspase-1 activity. Active caspase-1 cleaves the Z-WEHD-aminoluciferin substrate, leading to the release of aminoluciferin, resulting in a luciferase reaction and light production, which can be measured with a luminescence microplate reader [34].

**Figure 4:**
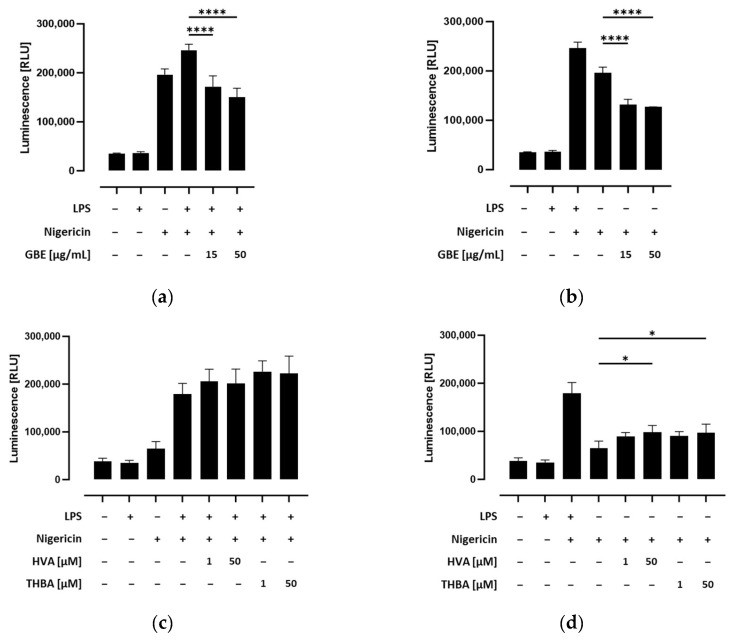
Effect of grape/blueberry anthocyanins and their gut-derived metabolites on caspase-1 activity in THP-cells. THP-1 monocytes were preincubated with the indicated concentrations of grape/blueberry anthocyanins and their gut-derived metabolites before the NLRP3 inflammasome was activated as mentioned in the methods section. Caspase-1 activity was measured by using the Caspase-Glo^®^ 1 Inflammasome Assay and luminescence was measured. Data are presented as mean ± SD (*n* = 3). Significant differences compared to (**a**,**c**) LPS- and nigericin-stimulated cells or (**b**,**d**) cells treated only with nigericin were calculated using one-way ANOVA followed by Dunnett’s multiple comparisons test. * *p* < 0.05, **** *p* < 0.0001. RLU, relative light unit.

As shown in Figure S7a, inflammasome activation led to a significant increase in caspase-1 activity in nigericin-stimulated THP-1 monocytes. Nevertheless, this response was significantly enhanced by priming with LPS. To confirm the specific determination of caspase-1 activity, the caspase-1 inhibitor Ac-YVAD-CHO was used for testing in parallel wells in preliminary tests. We further assessed whether caspase-1 activity was influenced by grape/blueberry anthocyanins and their gut-derived metabolites in THP-1 cells. Therefore, THP-1 monocytes were preincubated with different concentrations of the GBE, HVA, or THBA before the NLRP3 inflammasome was activated by LPS and/or nigericin. We found that caspase-1 activity was significantly inhibited by preincubation with the GBE both in LPS and nigericin, as well as in cells treated only with nigericin (*p* < 0.0001; Figure 4a,b). Surprisingly, caspase-1 activity slightly but not significantly increased in LPS-primed cells preincubated with the gut-derived anthocyanin metabolites HVA and THBA (Figure 4c). Similarly, after inflammasome activation, caspase-1 activity was enhanced in unprimed HVA or THBA-treated THP-1 monocytes (*p* < 0.05; Figure 4d). In conclusion, these results indicate that grape/blueberry anthocyanins inhibit inflammasome activation by inhibiting caspase-1 activity, while gut-derived metabolites may exert contrary effects on caspase-1-activity in THP-1 monocytes.

### 3.6. Grape/Blueberry Anthocyanins and Their Gut-Derived Metabolites Ameliorate Inflammatory Cytokine Secretion after NLRP3 Inflammasome Activation in THP-1 Monocytes

**Figure 5:**
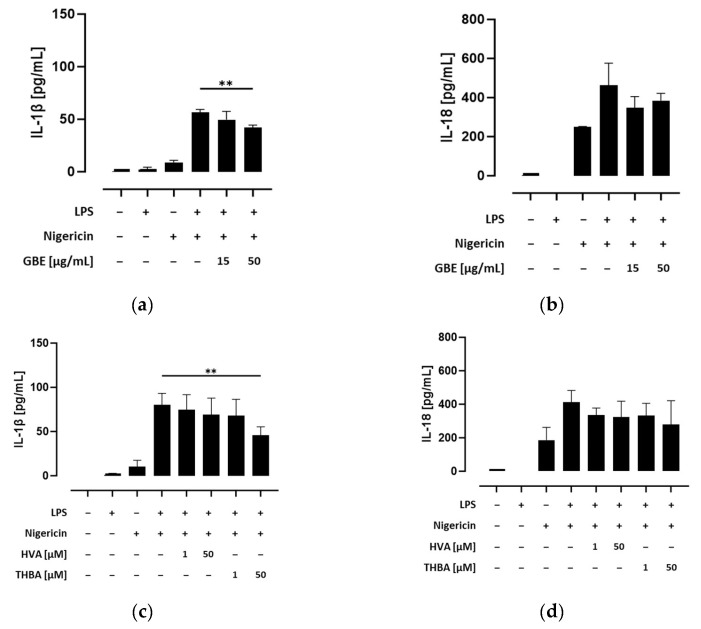
Effect of grape/blueberry anthocyanins and their gut-derived metabolites on inflammatory cytokine secretion in THP-1 cells. THP-1 monocytes were preincubated with the indicated concentrations of grape/blueberry anthocyanins and their gut-derived metabolites before the NLRP3 inflammasome was activated as mentioned in the methods section. The release of (**a**,**c**) IL-1β and (**b**,**d**) IL-18 into the cell-culture supernatant was measured using ELISA. Data are presented as mean ± SD of at least three replicated experiments. Significant differences compared to LPS- and nigericin-stimulated cells were calculated using one-way ANOVA followed by Dunnett’s multiple comparisons test. ** *p* < 0.01.

Secretion of the inflammatory cytokines IL-1β and IL-18 requires the cleavage of their pro-forms into their bioactive forms by active caspase-1 [21]. Thus, we next investigated the efficacy of grape/blueberry anthocyanins and their gut-derived metabolites to attenuate pro-inflammatory cytokine release. Therefore, THP-1 monocytes were preincubated with different concentrations of the GBE, HVA, or THBA before the NLRP3 inflammasome was activated by LPS and/or nigericin, and IL-1β and IL-18 concentrations in the cell-culture supernatants were measured using ELISAs. Our results show that NLRP3 inflammasome activation significantly induced IL-1β and IL-18 secretion in primed and unprimed THP-1 monocytes (Figure S7b,c), albeit that inflammatory cytokine release was significantly enhanced by LPS priming. However, preincubation with the GBE (15 and 50 μg/mL) decreased IL-1β concentrations in the cell-culture supernatants of LPS- and nigericin-treated cells, whereas the effect of the higher concentration was still significant after correction for multiple testing (*p* < 0.01; Figure 5a). In addition, preincubation with THBA (50 μM) drastically declined IL-1β secretion after LPS/nigericin-induced inflammasome activation (*p* < 0.01), whereas preincubation with HVA showed only modest effects (Figure 5c). In addition, the efficacy of grape/blueberry anthocyanins and their gut-derived metabolites to attenuate IL-18 secretion was less pronounced even at high concentrations (Figure 5b,d). After inflammasome activation, IL-18 release by THP-1 monocytes was decreased by preincubation with the GBE (15 μg/mL) and THBA (50 μM). However, after correction for multiple testing these effects were no longer statistically significant. Consequently, these results indicate that grape/blueberry anthocyanins and their gut-derived metabolites ameliorate inflammatory cytokine secretion after inflammasome activation in THP-1 monocytes, whereas the efficacy of grape/blueberry anthocyanins and their metabolites to attenuate IL-18 secretion is less pronounced.

## 4. Discussion

In the last years, growing evidence indicates a link between excessive NLRP3 inflammasome activation and inflammatory diseases [21]. Therefore, searching for new approaches to preventing NLRP3 inflammasome activation is crucial. Anthocyanins and their gut-derived metabolites are known for their anti-inflammatory and anti-oxidative properties [23,24,25]. Therefore, the present study aimed to determine the potential of grape/blueberry anthocyanins, and especially their gut-derived metabolites, to attenuate NLRP3 inflammasome activation.

Canonical inflammasome activation is a two-step process comprising priming and activation. During the priming step, damage-associated molecular patterns and pathogen-associated molecular patterns such as LPS induce the nuclear factor (NF)-κB mediated expression of different NLRP3 inflammasome components [35]. For this reason, we primed THP-1 monocytes with LPS for several hours before ASC and NLRP3 protein expression was assessed using flow cytometry. We noted a high basal NLRP3 protein expression, which was also observed by Gritsenko et al. [19]. However, neither ASC nor NLRP3 protein expression was induced by LPS in the present study. In another study, ASC protein expression was not significantly altered by LPS priming in primary human monocytes, whereas NLRP3 protein expression was significantly upregulated [36]. In addition, LPS priming of primary human monocytes and THP-1 cells also resulted in higher NLRP3 protein expression rates in a further study [19]. However, in these studies, 10- to 100-fold higher LPS concentrations were used, and protein expression was assessed through western blot analyses [19,36]. In contrast, protein expression was determined using flow cytometry on a single cell level in the present study. Therefore, discrepancies in study results may be due to experimental and methodological differences.

Several studies reveal that anthocyanins exert anti-inflammatory properties by inhibiting NF-κB signaling pathway activation [37]. Therefore, we next examined the influence of grape/blueberry anthocyanins and their gut-derived metabolites on ASC and NLRP3 protein expression in THP-1 monocytes. A recent study reported that ASC and NLRP3 mRNA levels were significantly decreased by different blueberry extracts in LPS-treated murine RAW264.7 macrophages [38]. Moreover, NLRP3 protein expression was also significantly inhibited to varying extents depending on the respective blueberry variety [38]. In other studies, malvidin, which is the predominating anthocyanin in blueberries [39], inhibited LPS-induced NLRP3 protein expression in various mouse tissues [26,27,40,41]. In contrast, the observed effects of malvidin on ASC protein expression are more inconsistent. While LPS-induced ASC protein expression has been found to be significantly decreased by malvidin treatment in liver and kidney tissues of mice, ASC expression in mice cerebrum was not affected [26,27,40]. Similarly, in a randomized placebo-controlled clinical trial, mRNA levels of ASC and NLRP3 were not significantly lower in peripheral blood mononuclear cells (PBMCs) from patients with nonalcoholic fatty liver disease (NAFLD), compared to the control group, after 12 weeks of anthocyanin supplementation [42]. In addition, no inhibitory effects of grape/blueberry anthocyanins and their gut-derived metabolites on ASC or NLRP3 protein expression in THP-1 monocytes were observed in the present study. Therefore, we next investigated whether grape/blueberry anthocyanins and their gut-derived metabolites inhibit NLRP3 inflammasome assembly rather than ASC or NLRP3 expression.

ASC speck formation is a unique hallmark of inflammasome activation that can be observed using fluorescence microscopy. In addition, western blot analyses can be used to discriminate between ASC monomers and ASC oligomers [22]. However, these techniques are either non-quantitative or time consuming. Therefore, ASC speck formation was determined using intracellular flow cytometry in the present study. This powerful approach was recently introduced by Sester et al. and allows researchers to assess the formation of ASC specks on a single cell level [22,31]. Although neither ASC nor NLRP3 protein expression was induced by LPS priming in the present study, inflammasome activation was significantly higher in LPS-primed cells compared to unprimed cells, as shown by a significantly higher percentage of ASC speck-positive THP-1 monocytes. Therefore, our results illustrate that priming regulates NLRP3 inflammasome activation beyond the transcriptional upregulation of inflammasome components. Indeed, growing evidence indicates that priming of the NLRP3 inflammasome induces complex post-translational modifications such as ubiquitination and phosphorylation, which influence NLRP3 inflammasome activation [21,43]. To the best of our knowledge, our results show for the first time that ASC speck formation is attenuated by grape/blueberry anthocyanins and their gut-derived metabolites in primed, as well as unprimed, THP-1 monocytes. The fact, that ASC speck formation was also diminished in unprimed monocytes indicates that anthocyanins and their gut-derived metabolites may possibly prevent NLRP3 oligomerization and/or ASC-NLRP3 interaction. In this context, Erianin, a low-molecular-weight molecule with two phenyl rings, has been reported to prevent NLRP3 inflammasome assembly by direct binding to the NLRP3 protein [44]. On the other hand, nigericin treatment causes mitochondrial dysfunction, resulting in increased reactive oxygen species (ROS) generation and oxidative stress, which are known to induce NLRP3 inflammasome activation [32]. Cellular redox homeostasis is maintained by the nuclear factor erythroid 2-like factor 2 (Nrf2) signaling pathway [45]. A recent study reported that malvidin significantly increased Nrf2 protein expression in the colon tissue of mice with LPS-induced septic intestinal injury [41]. Furthermore, another study found that pretreatment with malvidin decreased LPS/ATP-induced ASC oligomerization, as well as ASC speck formation, in human renal tubular epithelial cells (HK-2 cells) by activating the Nrf2 pathway, and decreased ROS generation [27]. In addition, malvidin has been found to alleviate LPS/ATP-induced mitochondrial dysfunction and ROS generation in BV-2 microglia cells [40]. Similarly, anthocyanins from *Hibiscus syriacus* L. inhibited LPS/ATP-induced NLRP3 inflammasome activation in BV-2 microglia cells by alleviating mitochondrial ROS production in a further study [46]. Therefore, grape/blueberry anthocyanins and their gut-derived metabolites may inhibit ASC speck formation in THP-1 monocytes by ameliorating oxidative stress and preventing the interactions of inflammasome components.

Upon NLRP3 inflammasome activation, ASC and pro-caspase-1 molecules interact via their respective caspase recruitment domains, leading to the auto-proteolytic activation of caspase-1, which is an inflammatory caspase that catalyzes the maturation and release of IL-1β and IL-18 [32]. Hence, inhibition of caspase-1 activity may diminish the inflammatory response. Therefore, we next investigated whether the observed reduction in ASC speck formation by grape/blueberry anthocyanins and their gut-derived metabolites was accompanied by a decrease in capase-1 activity. Our results show that caspase-1 activity was significantly inhibited by grape/blueberry anthocyanins in primed and unprimed THP-1 monocytes, indicating that grape/blueberry anthocyanins may inhibit the recruitment and/or the auto-catalytic activation of caspase-1. However, anthocyanins may also inhibit caspase-1 expression. A recent study reported that treatment with different blueberry extracts reduced the levels of mRNA and protein expression of caspase-1 in RAW264.7 macrophages [38]. In addition, studies have found LPS-induced protein expression of cleaved caspase-1 to be significantly decreased by malvidin treatment in several mouse tissues [26,27,40]. Similarly, malvidin reduced the protein levels of cleaved caspase-1 in LPS/ATP-treated HK-2 cells [27]. Therefore, grape/blueberry anthocyanins may decrease caspase-1 activity by inhibiting the expression and activation of its inactive pro-form.

The maturation and release of the inflammatory cytokines IL-1β and IL-18 require active caspase-1 [21]. Therefore, we finally examined the influence of grape/blueberry anthocyanins and their gut-derived metabolites on IL-1β and IL-18 secretion into the cell-culture supernatant of LPS- and nigericin-treated THP-1 monocytes. As recently reported, IL-1β is not basally expressed in human monocytes, whereas IL-18 is constitutively expressed [19]. However, in the present study NLRP3 inflammasome activation by nigericin treatment significantly induced IL-1β and IL-18 release in primed and unprimed THP-1 monocytes. These results indicate that at least low levels of IL-1β are basally expressed in THP-1 monocytes. In addition, preincubation with grape/blueberry anthocyanins and their gut-derived metabolites significantly decreased IL-1β concentrations in the cell-culture supernatants of LPS- and nigericin-treated THP-1 monocytes, whereas the effects on IL-18 concentrations were only modest and less pronounced even at high concentrations. Therefore, grape/blueberry anthocyanins and their gut-derived metabolites may not only attenuate ASC speck formation and caspase-1 activity but also pro-inflammatory cytokine expression. Indeed, gene expression of IL-1β has been found to be significantly inhibited by blueberry extracts in murine RAW264.7 macrophages [38], and red raspberry supplementation has been found to prevent high-fat-diet-induced IL-1β, as well as IL-18, mRNA and protein expression in the liver tissue of mice [47]. Similarly, in other studies, malvidin alleviated LPS-induced IL-1β expression in several mouse tissues [26,27,41]. In addition, after supplementation of anthocyanins for 12 weeks, IL-1β and IL-18 mRNA expression rates have been found to be significantly lower in PBMCs from NAFLD patients compared to the placebo group [42], and IL-1β and IL-18 plasma levels also significantly decreased in the anthocyanin group [42]. Taken together, this evidence indicates that grape/blueberry anthocyanins and their gut-derived metabolites attenuate pro-inflammatory cytokine release upon NLRP3 inflammasome activation and therefore may exert beneficial health effects in the prevention and treatment of inflammatory diseases.

To the best of our knowledge, our results show for the first time that grape/blueberry anthocyanins and their gut-derived metabolites exert anti-inflammatory effects by attenuating NLRP3 inflammasome activation in primed and unprimed human monocytes. In particular, THBA exhibited a strong potential to inhibit NLRP3 inflammasome activation, while the dampening impact of THBA on NLRP3 inflammasome activation was more pronounced at the high, supraphysiological concentration. In contrast, maximal systemic THBA concentrations between 103 and 582 nM were observed after single doses of 500 mg ^13^C-labeled cyanidin-3-glucoside or elderberry anthocyanins, respectively [15,16]. Therefore, the physiological relevance of the current results has to be considered. However, potential additive and synergistic effects of anthocyanin metabolites may exert in vivo [48], amplifying the efficiency of physiologically attainable anthocyanin and metabolite concentrations to inhibit inflammasome activation. One strength of the current study is that several events of the NLRP3 inflammasome activation cascade were monitored through the evaluation of multiple read-outs. In addition, ASC speck formation was determined using intracellular flow cytometry, which is a sensitive and powerful approach to assess inflammasome activation. However, the underlying molecular mechanisms by which grape/blueberry anthocyanins and their gut-derived metabolites attenuate NLRP3 inflammasome activation remain to be shown and should be addressed in future studies, since understanding the precise molecular pathways involved would provide deeper insights into the observed outcomes. One limitation of the present study is that cancerogenic cells, like THP-1 monocytes, may not completely reflect the behavior of primary cells. However, THP-1 cells display similar features of NLRP3 inflammasome activation compared to primary human monocytes. In addition, THP-1 cells have been confirmed as a sufficient model to assess NLRP3 inflammasome activation in prior studies [19,29]. Nevertheless, most of our knowledge about the influence of anthocyanins and their metabolites on NLRP3 inflammasome activation comes from preclinical in vitro studies and animal models. Translating findings from those models to humans requires careful consideration of interspecies differences in metabolism and physiology. Therefore, further clinical studies confirming these findings in primary human monocytes are necessary to provide conclusions that are even more definitive.

## 5. Conclusions

In conclusion, our results show that grape/blueberry anthocyanins and their metabolites exert anti-inflammatory effects by attenuating NLRP3 inflammasome activation. Therefore, grape/blueberry anthocyanins and especially their physiological relevant metabolite THBA might be useful for the prevention and treatment of several inflammatory diseases.

## Figures and Tables

**Table 1:** Anthocyanin composition of the powdered anthocyanin-rich grape/blueberry extract.

Anthocyanins ^1^	Rt [min]	[M+H]^+^	λmax [nm]	mg/g	[%]
Delphinidin-3,5-diglucoside	7.10	627, 465, 303	520	0.3	0.4
Cyanidin-3,5-diglucoside	8.65	611, 449, 287	514	1.3	2.0
Delphinidin-3-galactoside	9.57	465, 303	522	3.2	5.2
Delphinidin-3-glucoside	10.41	465, 303	522	3.7	6.0
Peonidin-3,5-diglucoside	11.13	625, 463, 301	513	23.4	38.1
Delphinidin-3-arabinoside	11.84	435, 303	522	6.7	10.9
Malvidin-3,5-diglucoside	12.06	655, 493, 331	521	6.7	10.9
Petunidin-3-galactoside	12.72	479, 317	524	0.8	1.3
Cyanidin-3-arabinoside	13.03	419, 287	517	1.5	2.5
Petunidin-3-glucoside	13.52	479, 317	521	2.6	4.3
Peonidin-3-galactoside	14.10	463, 301	515	0.2	0.4
Petunidin-3-arabinoside	14.59	449, 317	524	0.5	0.9
Peonidin-3-glucoside	15.00	463, 301	516	5.1	8.3
Malvidin-3-glucoside	15.95	493, 331	524	3.9	6.4
Malvidin-3-arabinoside	16.99	463, 331	526	0.3	0.5
Malvidin-3-(6″-coumaryl)-5-diglucoside	21.69	801, 639, 493, 331	524	1.2	1.9
∑				61.4	100

^1^ The powdered grape/blueberry extract was analyzed using HPLC–PDA/ESI–MS (*n* = 2), and the data were expressed as mean ± SD mg cyanidin-3-glucoside equivalents per g. Rt, retention time; [M+H]^+^, photoionization mass; λmax (lambda max), wavelength maxima.

## Data Availability

Data is contained within the article or Supplementary Materials.

## Supplementary Materials

The following supporting information can be downloaded at: https://www.mdpi.com/article/10.3390/metabo14040203/s1, Figure S1: Representative HPLC-MS chromatogram of the powdered anthocyanin-rich grape/blueberry extract; Figure S2: Molecular structures of gut-derived anthocyanin metabolites; Figure S3: Gating strategy to assess cell viability; Figure S4: Gating strategy to assess ASC and NLRP3 protein expression; Figure S5: Gating strategy to assess ASC speck formation; Figure S6: Effect of grape/blueberry anthocyanins and their gut-derived metabolites on ASC and NLRP3 protein expression in THP-1 cells; Figure S7: NLRP3 inflammasome activation in THP-1 cells.
