# Supplementary material for: Grape/Blueberry Anthocyanins and Their Gut-Derived Metabolites Attenuate LPS/Nigericin-Induced Inflammasome Activation by Inhibiting ASC Speck Formation in THP-1 Monocytes
Source: Metabolites. 2024 Apr 3;14(4):203. doi: 10.3390/metabo14040203 (PMC11051782; doi:10.3390/metabo14040203)
Supplement: Supplementary file 1 [file metabolites-14-00203-s001.zip › metabolites-2918095-supplementary.pdf]

Grape/Blueberry Anthocyanins and Their Gut-Derived Metabolites Attenuate LPS/Nigericin-Induced Inflammasome Activation by Inhibiting ASC Speck Formation in THP-1 Monocytes

Inken Behrendt [1,*], Isabella Röder [2], Frank Will [2], Gabriela Michel [3,4], Daniela Grote [1], Zoe Martin [1], Hanna Pauline Dötzer [1], Mathias Fasshauer [1], Martin Speckmann [3,4] and Sabine Kuntz [1]

[1] Institute of Nutritional Science, Justus-Liebig-University Giessen, 35390 Giessen, Germany; grote.d@outlook.de (D.G.); martin.zoe@gmx.de (Z.M.); hanna.doetzer@t-online.de (H.P.D.); mathias.fasshauer@ernaehrung.uni-giessen.de (M.F.); sabine.kuntz@nutr.jlug.de (S.K.)
[2] Department of Beverage Research, Hochschule Geisenheim University, 65366 Geisenheim, Germany; isabella.roeder@googlemail.com (I.R.); frank.will@hs-gm.de (F.W.)
[3] Institute for Clinical Immunology, Transfusion Medicine and Hemostaseology, Justus-Liebig-University Giessen, 35392 Giessen, Germany; gabriela.michel@immunologie.med.uni-giessen.de (G.M.); martin.speckmann@immunologie.med.uni-giessen.de (M.S.)
[4] Flow Cytometry Core Facility, Department of Medicine, Justus-Liebig-University Giessen, 35392 Giessen, Germany
* Correspondence: inken.behrendt@ernaehrung.uni-giessen.de; Tel.: +49 641 9939067

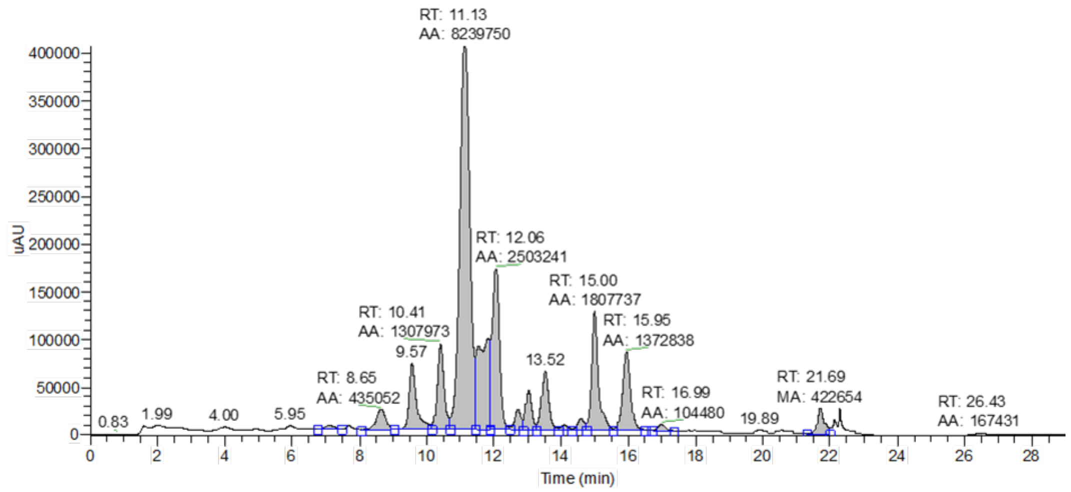

Figure S1. Representative HPLC-PDA/ESI-MS chromatogram of the powdered anthocyanin-rich grape/blueberry extract. Identified anthocyanins according to their retention times (RT): 7.10, Delphinidin-3,5-diglucoside; 8.65, Cyanidin-3,5-diglucoside; 9.57, Delphinidin-3-galactoside; 10.41, Delphinidin-3-glucoside; 11.13, Peonidin-3,5-diglucoside; 11.84, Delphinidin-3-arabinoside; 12.06, Malvidin-3,5-diglucoside; 12.72, Petunidin-3-galactoside; 13.03, Cyanidin-3-arabinoside; 13.52, Petunidin-3-glucoside; 14.10, Peonidin-3-galactoside; 14.59, Petunidin-3-arabinoside; 15.00, Peonidin-3-glucoside; 15.95, Malvidin-3-glucoside; 16.99, Malvidin-3-arabinoside; 21.69, Malvidin-3-(6''-coumaryl)-5-diglucoside.

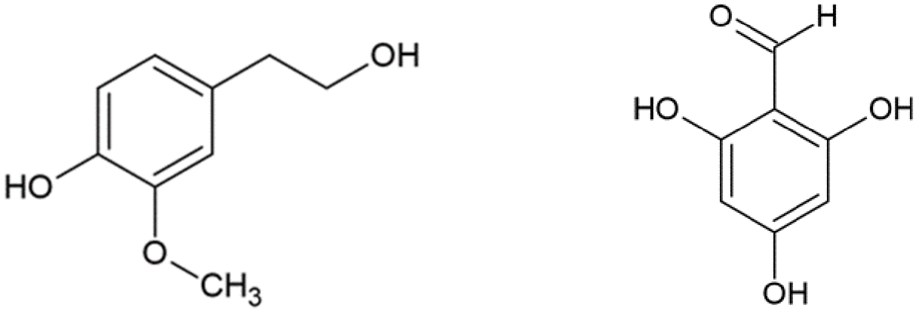

(**a**) (**b**)

Figure S2. Molecular structures of gut-derived anthocyanin metabolites. (**a**) Homovanillyl alcohol (HVA) and (**b**) 2,4,6-trihydroxybenzaldehyde (THBA). Molecular strtuctures were drawn with ChemSketch version 14.00, Advanced Chemistry Development, Inc. (ACD/Labs), Toronto, ON, Canada, www.acdlabs.com.

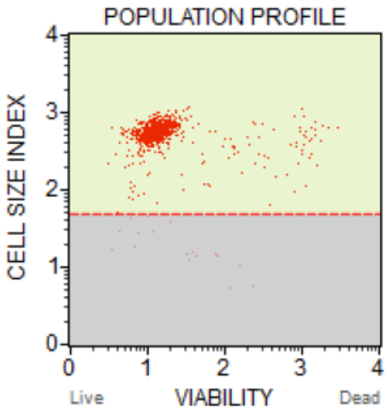

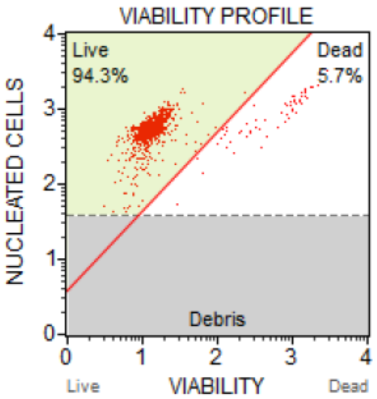

(**a**) (**b**)

Figure S3. Gating strategy to assess cell viability. (**a**) To exclude cellular debris cells were first gated based on their cellular size (horizontal line). (**b**) Then nucleated cells were gated (angled marker) for their staining with the membrane-permeant DNA staining dye, that stains all cells with a nucleus (nucleated cells), and a DNA-binding dye, that stains dead and dying cells which have lost their membrane integrity (viability), to discriminate live and dead cells.

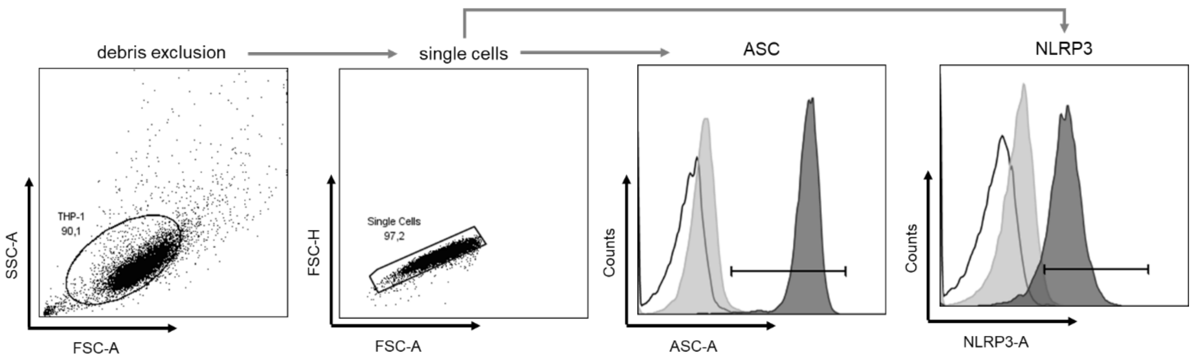

Figure S4. Gating strategy to assess ASC and NLRP3 protein expression. To exclude debris, cells were first gated using forward light scatter-area (FSC-A) versus side scatter-area (SSC-A). Next, FSC-A versus FSC-height (FSC-H) was used to perform doublet exclusion. Single cells were then gated for ASC and NLRP3 expression compared to the matching isotype control and median fluorescence intensity (MFI) was assessed. Open histogram unstained, light grey filled histogram matching isotype control, dark grey filled histogram stained.

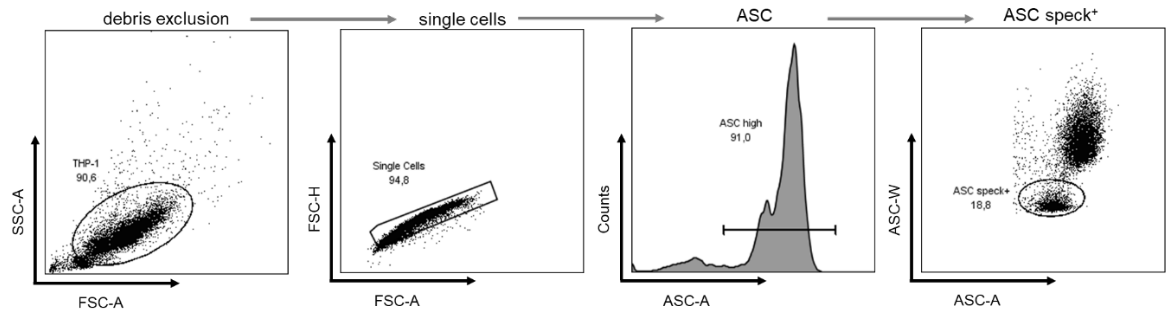

Figure S5. Gating strategy to assess ASC speck formation. ASC high expressing cells were gated as indicated in Figure S3. ASC high cells were further gated using ASC fluorescence pulse area (ASC-A) and ASC fluorescence pulse width (ASC-W). ASC speck forming cells were selected via the observed reduction in ASC-W and the percentage of ASC speck positive cells was quantified.

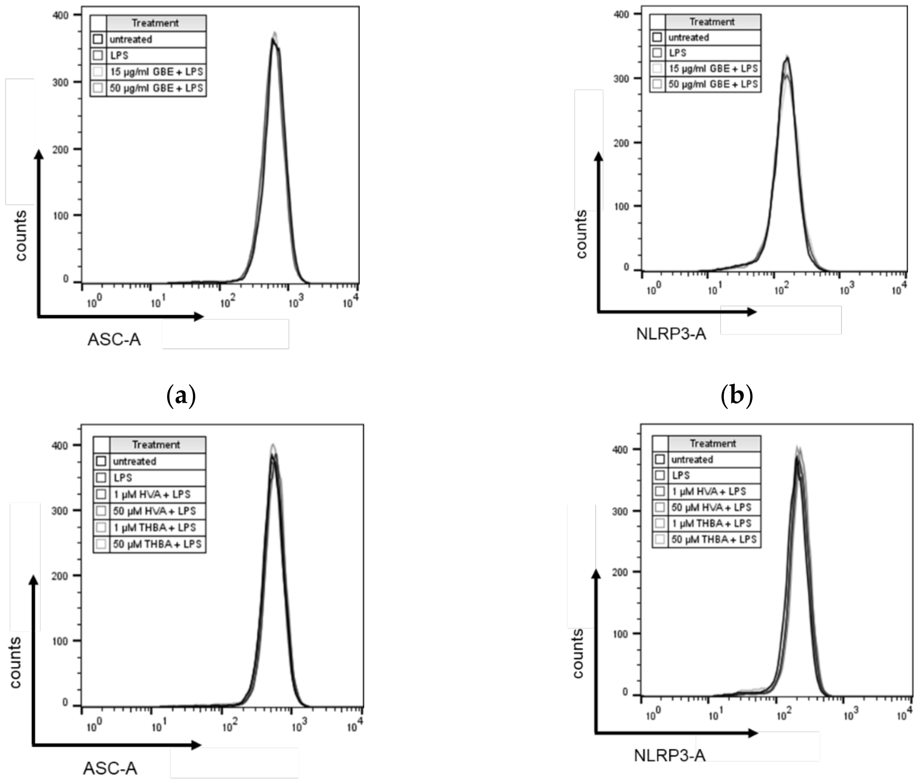

(**a**) (**b**)

(**c**) (**d**)

Figure S6. Effect of grape/blueberry anthocyanins and their gut-derived metabolites on ASC and NLRP3 protein expression in THP-1 cells. THP-1 monocytes were pretreated with the indicated concentrations of grape/blueberry anthocyanins and their gut-derived metabolites, before cells were primed with LPS as mentioned in the methods section. Protein expression of (**a,c**) ASC and (**b,d**) NLRP3 was assessed as median fluorescence intensity (MFI) by intracellular flow cytometry. The presented histogram overlays depict representative results from one of at least three replicated experiments.

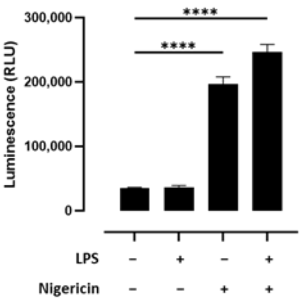

(**a**)

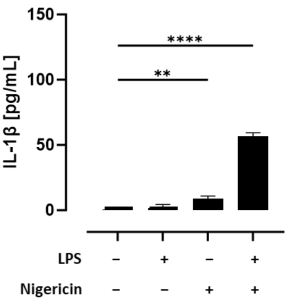

(**b**)

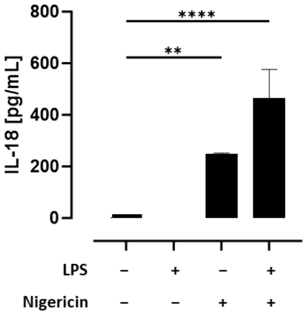

(**c**)

Figure S7. NLRP3 inflammasome activation in THP-1 cells. THP-1 monocytes were left untreated or primed with 10 ng/ml LPS for 4 h before the inflammasome was activated by adding 10 µM nigericin for further 40 min. (**a**) Caspase-1 activity was measured by using the Caspase-Glo® 1 Inflammasome Assay and luminescence was measured. Release of (**b**) IL-1β and (**c**) IL-18 into the cell culture supernatant were measured by ELISAs. Data are presented mean ± SD of at least three replicated experiments. Significant differences compared to the untreated control were calculated using one-way ANOVA, followed by Dunnett's multiple comparison test. **$p < 0.01$ and ****$p < 0.0001$. RLU, relative light unit. RLU, relative light unit.
